# Serum Oxidative and Nitrosative Stress Markers in Clear Cell Renal Cell Carcinoma

**DOI:** 10.3390/cancers15153995

**Published:** 2023-08-07

**Authors:** Sabina Galiniak, Marek Biesiadecki, Mateusz Mołoń, Patrycja Olech, Krzysztof Balawender

**Affiliations:** 1 Institute of Medical Sciences, Medical College, Rzeszow University, Warzywna 1a, 35-310 Rzeszow, Poland; kbalawender@ur.edu.pl; 2 Institute of Biology, College of Natural Sciences, Rzeszow University, Zelwerowicza 4, 35-601 Rzeszow, Poland

**Keywords:** clear cancer renal cell carcinoma, protein oxidation, 3-nitrotyrosine, serum total antioxidant capacity

## Abstract

**Simple Summary:**

Oxidative stress, defined as a disturbance in the balance between the production of reactive oxygen species and antioxidant defences, is discussed in relation to its possible role in many pathological conditions, including carcinogenesis. The identification of the oxidative and nitrosative modification of proteins and the definition of their roles in clear cell renal cell carcinoma may be helpful in the elaboration of targeted therapeutic approaches to mitigate protein damage. Therefore, this study aimed to investigate the status of oxidative/nitrosative stress and to explore its role in the development and progression. Oxidative stress increased with the progression of the disease assessed according to the TNM classification and histological grade. Moreover, the presence of angioinvasion in the patients affected the level of nitrite/nitrate, lipid peroxidation and total antioxidant capacity of serum. This is the first report presenting the level of oxidative/nitrosative stress markers among clear cell renal cell carcinoma patients with and without angioinvasion.

**Abstract:**

Oxidative stress is believed to be a factor in the development and progression of renal cell carcinoma (RCC). The identification of the oxidative and nitrosative modification of proteins and the definition of their roles in clear cell RCC (ccRCC) may be helpful in the elaboration of targeted therapeutic approaches to mitigate protein damage. This study aimed to investigate the status of oxidative/nitrosative stress and to explore its role in the development and progression. The studied group consisted of 48 newly diagnosed ccRCC and 30 healthy controls. Serum levels of oxidative stress markers—advanced oxidation protein products (AOPP), thiol groups, Amadori reaction products, 3-nitrotyrosine, nitrate/nitrite, malondialdehyde (MDA), 4-hydroxy-2-nonenal and total antioxidant capacity (TAC)—were determined. Additionally, associations between tumour stage assessed according to TNM classification, histological grade, and the effect of the presence of angioinvasion on the level of stress markers were evaluated. The levels of Amadori products, 3-nitrotyrosine, and nitrate/nitrite were elevated, while the levels of thiol groups and TAC decreased in the ccRCC group. The levels of AOPP, Amadori, and 3-nitrotyrosine increased, and thiol groups and TAC levels decreased with the increasing pathological stage of the tumour. In the case of advanced histological assessment of the tumour, we found decreasing levels of thiol groups and increasing levels of MDA. In patients with angioinvasion, nitrate/nitrite and MDA levels were significantly elevated compared to those in patients without angioinvasion. Oxidative stress increased with the progression of the disease assessed according to the TNM and histological grade. These results demonstrate systemic oxidative stress in ccRCC, suggesting the therapeutic application of antioxidants.

## 1. Introduction

Renal cell carcinoma (RCC) represents approximately 2.4% of all malignancies in adults, with the highest incidence occurring in Western countries and nearly 270,000 new cases diagnosed annually worldwide [1,2,3]. It is generally reported that there has been an annual increase in incidence of 2% both globally and in Europe over the past two decades, leading to an estimated 99,200 new cases of RCC within the European Union in 2018 [4,5]. Epidemiologically, it is the seventh most common kind of cancer in men and the ninth most common in women [6]. More than 60% of RCC diagnoses are made in men, with a higher incidence among older people. Among the identified risk factors, the most significant are obesity (hazard ratio [HR]: 1.71), hypertension (HR: 1.70) and smoking. About 50% of patients with RCC are current or former smokers [5,7]. The published meta-analysis reported that a pooled relative risk of incidence is 1.31 (95% confidence interval [CI], 1.22–1.40) for all smokers, 1.36 (95% CI, 1.19–1.56) for current smokers and 1.16 (95% CI, 1.08–1.25) for former smokers [8,9]. The RCC encompasses a broad spectrum of histopathological entities described in the World Health Organisation (WHO) classification for 2022. Three main types of RCC can be distinguished: clear cell (ccRCC), papillary (pRCC), and chromophobe (chRCC). In total, 75% of all RCC represent a clear cell subtype. Moreover, pRCC and chRCC represent the two most common non-ccRCC histological subtypes of renal cancer (13–20% and 5–7% of cases, respectively). However, it should be noted that the list of RCC included in the WHO 5th edition is close to 15 types [9,10]. Approximately, 2–3% of all RCCs are hereditary and several autosomal dominant syndromes have been identified, each with a distinct genetic basis and phenotype, with the most common one being Von Hippel Lindau disease [11,12].

Oxidative stress, defined as a disturbance in the balance between the production of reactive oxygen species (ROS) and antioxidant defences, is discussed in relation to its possible role in many pathological conditions, including carcinogenesis. ROS is a collective term to include superoxide (O_2_^▪−^), hydrogen peroxide (H_2_O_2_), hydroxyl radical (OH^▪^), singlet oxygen (^1^O_2_), peroxyl radical (LOO^▪^), alkoxyl radical (LO^▪^), and ozone (O_3_), and others [13]. Endogenous sources, generated during normal metabolism, include different cell organelles, such as mitochondria, peroxisomes and endoplasmic reticulum. On the other hand, exogenous sources include radiation X-rays, γ-rays, ultraviolet A, visible light in the presence of a sensitizer, chemical reagents, high temperatures, environmental pollutants, microbial infections, drugs and their metabolites. Moreover, accumulating evidence shows an important role for smoking-induced ROS and the resulting oxidative stress [14]. Increased levels of ROS are thought to be oncogenic, inducing damage to DNA, proteins and lipids, promoting genetic instability and tumourigenesis. Through its effect on signal transduction pathways, ROS increases cell proliferation, survival, and cellular migration [15,16]. The role of oxidative stress in the course of urogenital cancers is widely accepted [17,18,19]. The intense metabolic activity of tubular epithelial cells, glomerular cells, and activated macrophages makes them the main generators of ROS in the human kidneys [20,21]. Moreover, a recent study by Mahalingaiah et al. suggests that epigenetic reprogramming induced by low levels of chronic oxidative stress acts as a driving force for malignant transformation of kidney epithelial cells [22]. This study aimed to investigate the status of oxidative/nitrosative stress by determining serum levels of advanced oxidation protein products (AOPP), thiol groups, Amadori products, the ability of human serum albumin to bind cobalt, 3-nitrotyrosine, nitrate/nitrite as markers of protein oxidative/nitrosative damages, malondialdehyde (MDA) and 4-hydroxy-2-nonenal (4-HNE) levels as markers of lipid peroxidation, as well as non-enzymatic total antioxidant capacity (TAC), to explore the role of oxidative/nitrosative stress in the development and progression of cancer.

To our knowledge, this is also the first report that presents the level of AOPP, 4-HNE, and the ability of human serum albumin to bind cobalt in patients with RCC. Moreover, this is the first report presenting the level of oxidative/nitrosative stress markers among ccRCC patients with and without angioinvasion.

## 2. Materials and Methods

### 2.1. Ethical Issues

This study received approval from the Bioethics Committee of the Rzeszów University (Resolution No. 2022/037 and 2022/090). This study was performed in accordance with the guidelines for Good Clinical Practice and the Declaration of Helsinki.

### 2.2. Study Group

A single-centre cross-sectional study was performed in a sample of 48 patients with ccRCC and control subjects (n = 30). The participants were recruited from the Clinical Department of Urology and Urological Oncology of the Municipal Hospital in Rzeszów between 1 June 2021 and 30 September 2022.

Inclusion criteria: The study population consisted of patients with a primary renal tumour diagnosed via imaging (CT or MRI) who underwent surgery (laparoscopic nephron-sparing surgery or nephrectomy, depending on the stage of the tumour). Renal cancer was staged according to the TNM (tumour, node, metastasis) classification [23]. Tumours were graded according to the WHO/ISUP classification [24]. All patients in the study group were in stage cT1–3N0M0 at the time of study enrolment, and the imaging studies performed did not reveal lymph node metastasis or other metastases. The presence of angioinvasion was assessed by an experienced histopathologist.

**Figure 1:**
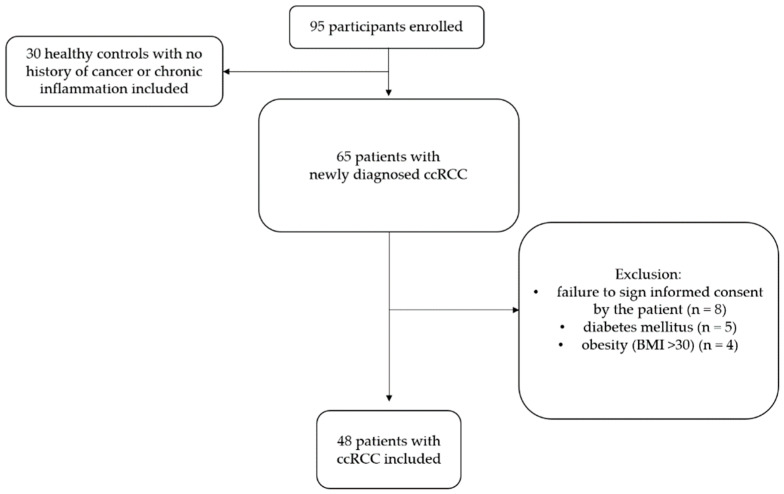
Flowchart of the participant recruitment process.

The exclusion criteria were the following: refusal to participate in the study or failure to sign informed consent; stage N+ or M+ malignancy according to the classification of TNM at the time of eligibility for the study; a history of other malignant, endocrine, infectious or inflammatory diseases, diabetes mellitus, dyslipidaemia or obesity. The T4 group has been dropped due to the very small group of patients operated on during the period analysed or disease progression beyond the N0M0 criteria. Figure 1 shows a flowchart of the participant recruitment process.

Healthy subjects were enrolled among people who came to the local clinic for check-ups from April to August 2022. Healthy participants had no history of cancer or other disease and had not taken any drugs, including supplements, for 30 days prior to the study. All volunteers in the control group underwent a standard urine test. All participants had similar socioeconomic status and food preferences at the time of medical interview.

### 2.3. Materials

All chemical reagents were of analytical grade, obtained from commercial suppliers (Sigma-Aldrich, Poznan, Poland). The 3-nitrotyrosine enzyme-linked immunosorbent assay (ELISA) kit was purchased from Immunodiagnostik AG (Bensheim, Germany). The nitrate/nitrite colorimetric assay kit was supplied by Cayman (Ann Arbor, MI, USA). The ELISA kit for non-enzyme 4-hydroxy-4-HNE was purchased from Wuhan Fine Biotech Co., Ltd. (Wuhan, China). Absorptiometric measurements were conducted in a Tecan Infinite 200 PRO multimode microplate reader (Tecan Group Ltd.; Männedorf, Switzerland). Measurements were made in triplicate, unless otherwise indicated.

### 2.4. Blood Sampling

Blood samples were collected according to standard procedure. Next, the samples were incubated at room temperature and centrifuged (1000× *g*, 10 min, 4 °C). The serum was aliquoted and stored at −80 °C until further analysis.

### 2.5. Blood Analysis

Complete blood counts, serum glucose, creatinine, urea, potassium, coagulological determinations, international normalised ratio (INR), and activated partial thromboplastin time (APTT) were analysed using standard laboratory methods and the results of these measurements were collected from the participants’ hospital records.

### 2.6. Biochemical Procedures

#### 2.6.1. Protein Assay

The protein concentration was estimated using the method of Lowry et al. [25]. Briefly, 250 μL of the Lowry reagent (formed by mixing 30 mL of 2% Na_2_CO_3_ in 0.1 M NaOH, 0.6 mL of 5% C_4_H_4_O_6_KNa⋅4H_2_O, and 0.6 mL of 2% CuSO_4_) was applied to a 96-well plate. Then, 50 μL of diluted serum was applied to each well, mixed, and incubated at room temperature for 10 min. Finally, 25 μL of the Folin–Ciocalteu reagent was added, mixed, and incubated at room temperature for 30 min. The absorbance was measured at a wavelength of 750 nm.

#### 2.6.2. AOPP Assay

The advanced oxidation protein products (AOPP) were estimated using the previously published method [26]. A total of 200 μL of serum diluted 1:5 with phosphate-buffered saline (PBS) was applied to the wells of a 96-well plate and 20 μL of acetic acid was added to each well. Absorbance was measured at 340 nm against a blank. The calibration curve was prepared using chlora-mine-T at concentrations of 0–100 μmol/L. AOPP concentration is expressed in nmol chloramine-T equivalents/mg protein.

#### 2.6.3. Thiol Group Assay

The content of thiol groups was estimated using the method of Ellman [27]. In each well of a 96-well plate, 20 μL of serum and 2 μL of 5,5′-dithiobis-(2-nitrobenzoic acid) (10 mg/mL of 0.1 M phosphate buffer, pH 8.0) were added to 100 μL 0.1 M phosphate buffer, pH 8.0. The samples were incubated in the dark at 37 °C for 1 h and the absorbance was measured at 412 nm against a blank. The thiol group content was calculated on the basis of a standard curve using glutathione as a standard.

#### 2.6.4. Characterization of Amadori Product via the NBT Assay

The content of the Amadori product was estimated using the previously published method [28] with nitro blue tetrazolium (NBT). Briefly, 100 μL aliquots of serum were added to the wells of a 96-well plate, followed by 100 μL of the NBT reagent (250 µmol/L in 0.1 M carbonate buffer, pH 10.35) and the plate was incubated at 37 °C for 2 h. The absorbance was measured at a wavelength of 525 nm. The Amadori products were estimated using an extinction coefficient of 12,640 M^−1^ cm^−1^ for monoformazan [29].

#### 2.6.5. Albumin Cobalt Binding (ACB) Assay

The ACB levels were measured via spectrophotometry using Bar-Or’s method [30]. The assay method involved adding 25 μL of 0.1% cobalt chloride to 100 μL of serum, gently mixing, and waiting 10 min for adequate cobalt–albumin binding. Then, 25 μL of di-thiothreitol (1.5 mg/mL) was added as a colorizing agent and the reaction was stopped 2 min later by adding 0.5 mL of 0.9% NaCl. Using a spectrophotometer at 470 nm, colour development with DTT was compared to a serum–cobalt blank without DTT and reported in absorbance units (ABSU). The measurements were carried out in duplicate.

#### 2.6.6. 3-Nitrotyrosine Assay

The 3-nitrotyrosine concentration was assessed with the 3-nitrotyrosine ELISA kit (Immundiagnostik AG, Bensheim, Germany), according to the provided protocol.

#### 2.6.7. Nitrate/Nitrite Assay

The nitrate/nitrite concentration was evaluated Using a nitrate/nitrite colorimetric assay kit (Cayman, Ann Arbor, MI, USA), according to the provided protocol.

#### 2.6.8. Total Antioxidant Capacity (TAC) Measured using the Method with ABTS and FRAP

ABTS^•^ (2,2′-azino-bis (3-ethylbenzothiazoline-6-sulphonic acid)) was formed by the reaction of 7 mM of ABTS solution with 2.45 mM of potassium persulfate, incubated for 24 h at room temperature and protected from light. Briefly, appropriate amounts of serum samples were added to a ABTS^•^ solution, and diluted such that 200 µL of the solution had an absorbance of 1.0 ± 0.04 in a well. The absorbance reading was carried out at 734 nm after six minutes of reaction at room temperature [31]. FRAP was determined colourimetrically by measuring the ferric reducing capacity of samples with 0.3 M acetate buffer (pH = 3.6), 0.01 M TPTZ (2,4,6-tripyridyl-s-triazine) in 0.04 M HCl and 0.02 M FeCl_3_·6H_2_O mixed in 10:1:1 and appropriate amounts of serum samples. Absorbance was carried out at 593 nm after 20 min incubation in room temperature [32]. Ethanol solutions with known concentrations of Trolox were used for calibration. The results were expressed in Trolox equivalents (μmol TE/L).

#### 2.6.9. MDA Assay

Serum samples were mixed with 200 μL of mixture (1:1) of 0.37% thiobarbituric acid (TBA) and 15% trichloroacetic acid in 0.25 M HCl to precipitate protein. The reaction was carried out at pH 2–3 at 100 °C for 40 min. The precipitate was pelleted by centrifugation and the absorption of the supernatants was read at a wavelength of 532 nm. The majority of TBA-reactive substances is MDA; therefore, the concentration of MDA in blood serum was expressed as μM MDA. The results were calculated using an absorption coefficient for MDA of 1.56 × 10^5^ M^−1^ cm^−1^ [33].

#### 2.6.10. 4-HNE Assay

The 4-HNE concentration was assessed with the 4-HNE ELISA kit (Wuhan Fine Biotech Co., Ltd.), according to the provided protocol.

### 2.7. Statistical Data Analysis

Data are expressed as mean values and standard deviations (SD). The Shapiro–Wilk test indicated that the variable had a normal distribution. Statistical significance of differences was determined using the Kruskal–Wallis test or Mann–Whitney U test. Statistical analysis of the data was performed with STATISTICA software package (version 13.3, StatSoft Inc. 2017, Tulsa, OK, USA).

## 3. Results

In total, 18 women and 30 men with ccRCC were recruited for this study. In addition, 30 healthy volunteers were enrolled in the control group. The basic characteristics and clinical laboratory values of the study participants are shown in Table 1.

There were no statistical differences in the basic characteristics between the study groups. The clinical haematological results were similar between the ccRCC group and healthy subjects. The study groups had similar clotting indices. There were no differences in serum creatinine, glucose and potassium levels between patients and healthy controls. Patients with ccRCC had elevated serum urea levels (*p* < 0.05). The TNM staging system was used to classify the extent of cancer spread. On this basis, the patients were divided into three subgroups based on the T category: T1–T3.

**Figure 2:**
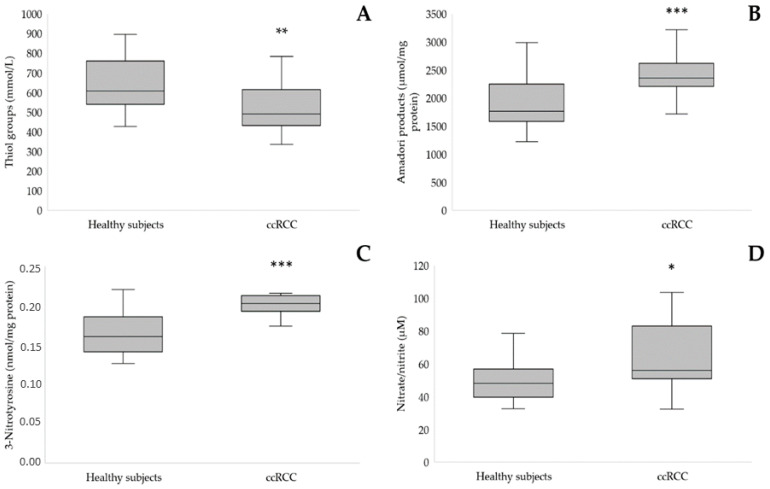
Comparison of thiol groups (**A**), Amadori products (**B**), 3-nitrotyrosine (**C**), and nitrate/nitrite (**D**) level in serum of patients with ccRCC as compared to the control group, * *p* < 0.05, ** *p* < 0.01, *** *p* < 0.001.

In our study, we observed a similar level of serum AOPP, one of the most frequently determined markers of protein oxidative modification of patients with ccRCC compared to the control group (Table 2). However, we found a significantly decreased level of thiol groups in the serum of participants with ccRCC (516.57 ± 114.3 vs. 649.88 ± 147.8 mmol/L, *p* = 0.005, Figure 2A). Similarly, another protein modification was observed—Amadori products were also higher in the serum of patients than healthy participants (*p* < 0.001, Figure 2B). 

We also determined the ability of albumin to bind cobalt ions via the ACB test. We found that patients with ccRCC and healthy controls had similar ACB values of ACB (*p* = 0.968, Table 2). We also determined the level of 3-nitrotyrosine, one of the markers indicating the severity of nitrosative stress. Participants with ccRCC had a significantly higher level of 3-nitrotyrosine than healthy controls (0.21 ± 0.029 vs. 0.16 ± 0.026 nmol/mg of protein, *p* < 0.001, Figure 2D). Furthermore, the level of serum nitrate/nitrite was estimated. We found a higher level of nitrate/nitrite in the ccRCC group than in healthy controls (65.85 ± 21.35 vs. 51.87 ± 15.11 μM, *p* = 0.011).

Moreover, the serum TAC was estimated using two methods. TAC of blood serum measured via ABTS^•^ was significantly decreased in the ccRCC group with respect to healthy controls (269.29 ± 11.7 vs. 284.65 ± 16.5 μmol TE/L, *p* < 0.001). Nevertheless, the TAC measured via FRAP did not differ between the study groups (Table 2). Lipids are one of the main compounds that are easily damaged by ROS. To assess lipid damage, we determined two markers of lipid peroxidation. In our study, the MDA levels were similar between the studied groups. In addition, the 4-HNE level was slightly elevated, but the difference was not statistically significant (Table 2).

Next, we investigated the effect of tumour stage on oxidative stress markers. Table 3 shows differences in markers of oxidative stress in patients with ccRCC depending on the pathological stage according to the TNM classification.

We found significantly increased levels of AOPP, Amadori products and 3-nitrotyrosine in patients with T3 tumour as compared to the T1 and T2 groups (*p* = 0.009, *p* = 0.015, *p* = 0.014, respectively). Moreover, the concentration of thiol groups and TAC measured using ABTS^•^ was significantly decreased in patients with T3 tumour when compared to other participants from the ccRCC group (*p* = 0.003 and *p* < 0.001). There were no statistical differences in the levels of other studied markers.

Afterwards, we examined the level of biomarkers depending on the histological grade (Table 4).

The levels of the markers tested, with the exception of the thiol groups and MDA, were similar in all groups. The levels of the thiol groups decreased significantly in the G3 group compared to patients with G1 (*p* = 0.003). Likewise, the MDA concentration was significantly higher in participants G3 than in participants G1 and G2 (*p* = 0.043 and *p* = 0.01, respectively).

Finally, we investigated whether the presence of angioinvasion in ccRCC patients affects the level of oxidative stress. Table 5 shows the level of biomarkers in ccRCC patients with angioinvasion compared to RCC participants without angioinvasion.

We found that patients with ccRCC with confirmed angioinvasion had elevated levels of nitrite/nitrate and MDA compared to those without angioinvasion. Moreover, we observed a decrease in TAC measured via the ABTS^•^ method in patients with angioinvasion.

## 4. Discussion

The aetiology of RCC is multifactorial and, despite the knowledge of definite risk factors for RCC, there are still factors and processes for which the influence on the carcinogenesis of RCC has not been fully elucidated. Oxidative stress is one of the many factors related to the development of RCC, as well as other cancers [34,35]. It is known that oxidative stress in cancer cells is induced by many endogenous or exogenous pro-oxidant factors, such as hypoxia, inflammation, and numerous therapeutics [36]. The identification of oxidative and nitrosative modification of proteins and the definition of their roles in RCC could be helpful in designing targeted therapeutic treatment to mitigate the oxidative damage of these proteins. In addition, literature data on the involvement of ROS in different stages of RCC are incomplete, and many reports have inconsistent results.

We found no differences in the level of AOPP between the study groups. The AOPP level was also not related to the histological grade and the presence of angioinvasion. However, the level of AOPP was related to the pathological stage of ccRCC. Elevated levels of protein oxidation products—AOPP and carbonyl groups—have been shown to occur in patients with laryngeal cancer [37], thyroid cancer [38], and colorectal cancer [39]. Moreover, carbonylated proteins were significantly elevated in the ccRCC groups compared to those in the control group in the study by Ene et al. [40].

Functional thiol groups play an important role in protecting cells from oxidative damage caused by ROS. In our study, there were differences in the level of the thiol groups in patients with ccRCC compared to healthy participants. On the contrary, the study by Aldemir et al. reported that there were no significant differences between the RCC group and the control group in terms of the thiol groups [41]. However, native thiol and total thiols were significantly lower in ccRCC than in controls [40]. In addition to thiol groups, glutathione also acts as a detoxicant and antioxidant. Ganesamoni et al. observed a significant decrease in the glutathione level in patients with RCC compared to controls [42]. Moreover, we have shown that the level of thiol groups is dependent on the pathological stage and histological grade of the cancer. The glutathione level was also significantly lower for high-grade than low-grade RCC [40]. ccRCC cells can use the metabolite of glutamine and glutathione metabolite to neutralize ROS released from lipid peroxidation [43].

One mechanism of protein modification is the nonenzymatic reaction between sugars, mainly glucose, and lysyl side chain and N-terminal amino groups of proteins, resulting in the formation of advanced glycation end products (AGEs). Exposure to high glucose results in the binding of sugar to amino acids and the formation of reversible Amadori products that progress to stable covalent adducts designated AGEs [44]. Glucose-induced modifications of proteins significantly alter the mechanical properties of the kidney extracellular matrix and may be important in the progression of renal damage [45]. We found a significantly elevated level of Amadori products in patients with ccRCC compared to that in healthy controls. In addition, increasing levels of Amadori products were observed as the pathological stage increased. Histological grade and the presence of angioinvasion do not affect the level of this marker. The receptors for AGEs were noted more freqeuntly in ccRCC samples than in those of adjacent normal tissues in the study by Guo et al. [46]. Similarly, glycosylation of protein, estimated by sialic acid and orosomucoids levels in serum, was statistically significantly increased in the RCC group compared to that in the control group [40]. Methylglyoxal-induced AGEs interaction with their receptors (i.e., RAGE) enhances RCC cell proliferation and migration by inducing proliferating cell nuclear antigen, matrix metalloproteinases, and inhibiting apoptosis in RCC via the Akt and extracellular signal regulated kinase signalling pathways [47]. Moreover, a recent study showed that the administration of AGEs to mice manifested an epithelial–mesenchymal transition of podocytes, podocyte injury and impaired kidney function [48]. The binding of AGEs to RAGE leads to the activation of NADPH oxidase that enhances the production of ROS, which may induce cell dysfunction and pathophysiological effects [49].

The ACB test is used as a marker of ischemia and oxidative stress that originate as a consequence of tissue hypoxia [50]. We found no difference in the ability of albumin to bind cobalt ions between ccRCC patients and healthy controls. Additionally, the ACB test did not work as a marker of tumour progression, as it was not related to pathological stage, histological grade and angioinvasion. Modifications at the N-terminus of albumin, an important binding site for metals such as cobalt, can occur due to acetylation or amino acid deletion [51]. However, it seems that in kidney cancer, there is no modification of the albumin structure by oxidative stress, which changes the ion-binding sites.

The nitration of tyrosine residues by NO-derived molecules generates 3-nitrotyrosine in proteins. In our study, 3-nitrotyrosine was significantly elevated in patients with ccRCC compared to that in healthy controls. We also observed a difference in the level of this marker between patients with T1 and T3 tumours. Histological grade and angioinvasion did not significantly affect 3-nitrotyrosine levels. Similarly to our results, serum levels of 3-nitrotyrosine increased significantly in the RCC groups compared to the control group [40]. However, 3-nitrotyrosine was significantly associated with high-grade tumours (*p* = 0.001) in 138 renal cell carcinomas [52]. Furthermore, 3-nitrotyrosine levels were significantly associated with high-grade tumours, while ROS (*p* < 0.003) and NO (*p* < 0.011) were significantly higher in patients with high-grade than low-grade RCC [53]. The autocrine NO signalling pathway is conserved in benign tumours and lost in malignant tumours. In malignant tumours, protein tyrosine nitration appears to be caused by the generation of reactive nitric oxide species via inflammatory infiltration [54]. 

Alterations in parameters that are representative of nitrosative stress, such as circulating nitrite and nitrate, could also be detected. We found a significantly elevated level of nitrate/nitrite in patients with RCC compared to healthy controls. However, the pathological stage and grade had no impact on the level of nitrate/nitrite in ccRCC patients. Patients with angioinvasion had a significantly higher level of nitrate/nitrite than participants without angioinvasion. No significant differences were found in serum nitrite + nitrate levels between 26 RCC patients and 8 control subjects. Moreover, serum levels were lower in patients with grade 3 and 4 tumours than in those with grade 1 and 2 tumours, but this was not statistically significant [55].

In our study, serum TAC measured via ABTS^•^ was significantly decreased in the ccRCC group compared to that in healthy controls. In contrast, we observed a progressively lower TAC with increasing tumour stage and histological grade. It is noteworthy that the TAC measured via the FRAP method did not differ between cancer patients and healthy controls. It is worth noting that the obtained differences in the FRAP and ABTS^•^ tests result from their different mechanisms of these methods, because the FRAP method does not measure thiol groups, which are common in serum [56]. On the contrary, the study by Aldemir et al. reported that there were no statistically significant differences between the RCC group and the control group in terms of total oxidant status and TAC determined using a novel automated measurement method [41].

The serum lipid peroxidation was unchanged in the ccRCC group compared to the healthy subjects. However, MDA was significantly elevated in the G3 group compared to the G1 participants. Furthermore, angioinvasion significantly increased the concentration of this marker.

Similarly to our results, lipid peroxidation in tumour tissue increased significantly with the increasing grade of ccRCC (*p* < 0.04) [42]. It was hypothesised that the elevated lipid peroxidation of the proximal renal tubules in obese and hypertensive individuals is associated with a high risk of kidney cancer [57]. Moreover, a large amount of lipid can accumulate in the ccRCC, leading to a large amount of ROS depending on excessive production of lipid, which is the premise of ferroptosis, a recently described form of iron-dependent oxidative cell death [58].

Redox status changes in RCC as a consequence of reduced antioxidant capacity, together with altered expression of glutathione S-transferase α, may be important factors in tumour development and progression [59]. However, no differences in the activity of antioxidant enzymes such as catalase, paraoxonase-1 myeloperoxidase and ceruloplasmin were found in CCR and healthy controls, which suggests that none could find a marker to distinguish patients with RCC and healthy people [41]. The prognostic significance of antioxidant enzyme activities, MDA, and glutathione levels in patients with RCC was also not confirmed in a recent study [19]. It seems that one of the most obvious therapeutic strategies to restore homeostasis in RCC cells is to lower ROS by supplementing with antioxidants, for example, a vitamin. Many clinical trials have been conducted with antioxidant treatment; however, the results are inconsistent [60].

The analysis performed is a single-centre study; hence, the main limitation is the size of the study group. The study was limited to patients at stage T1–3N0M according to the TNM classification. Patients at the T4 stage were excluded due to the limited sample size or progression of the disease beyond N0M0 criteria. We also did not analyse the level of antioxidants and antioxidant enzymes.

## 5. Conclusions

In conclusion, in our cohort of patients, we observed elevated levels of oxidative and nitrosative stress markers in ccRCC patients. Oxidative stress increased with the progression of the disease assessed according to the TNM and histological grade. Moreover, the presence of angioinvasion in the patients affected the level of nitrite/nitrate, lipid peroxidation and TAC of serum. New research is required to provide more insights into cellular biology of RCC, aiming to search for prognostic markers for patients with ccRCC.

## Figures and Tables

**Table 1:** Summary table of participant characteristics *.

		Healthy Controls	ccRCC Group	*p*
n		30	48	
F/M		10/20	18/30	
Age (years)	mean ± SD	67.07 ± 7.32	68.11 ± 9.4	0.677
	range	54–80	50–82	
BMI (kg/m^2^)	mean ± SD	25.97 ± 2.73	27.23 ± 2.81	0.056
	range	19.05–29.7	20.57–29.96	
**Complete blood count**				
WBC (10^3^/µL)	mean ± SD	7.45 ± 1.56	7.21 ± 2.14	0.770
	range	4.9–10	3.8–11.4	
LYM (%)	mean ± SD	29.19 ± 4.43	24.74 ± 8.97	0.087
	range	25.1–38.9	12.6–41.7	
MONO (%)	mean ± SD	7.46 ± 1.6	8.58 ± 3.01	0.170
	range	4.2–10	4.3–17.8	
NEU (%)	mean ± SD	63.66 ± 5.75	64.3 ± 9.24	0.438
	range	50.4–70	45.9–78	
EOS (%)	mean ± SD	2.06 ± 1.25	1.56 ± 1.01	0.241
	range	1–4.9	0.5–4.5	
BASO (%)	mean ± SD	0.49 ± 0.23	0.5 ± 0.25	0.603
	range	0.2–1.2	0.04–1.1	
**Coagulological determinations**				
Prothrombin time (s)	mean ± SD	11.75 ± 0.57	12.01 ± 0.86	0.717
	range	10.4–12.5	10.7–14.5	
Prothrombin time (%)	mean ± SD	94.77 ± 11.18	95.11 ± 9.77	0.899
	range	80–119	72–113	
INR	mean ± SD	1.04 ± 0.09	1.03 ± 0.07	0.645
	range	0.9–1.2	0.9–1.2	
APTT (s)	mean ± SD	30.11 ± 3.56	32.53 ± 5.16	0.275
	range	25.4–36.8	25.1–43.9	
**Serum analysis**				
Creatinine (mg/dL)	mean ± SD	0.91 ± 0.15	1.19 ± 0.8	0.603
	range	0.63–1.15	0.56–3.44	
Glucose (mg/dL)	mean ± SD	98.62 ± 6.64	102.57 ± 7.76	0.179
	range	89–106	89–110	
Urea (mg/dL)	mean ± SD	33.25 ± 8.69	44.5 ± 17.89	0.032
	range	20–49	22–85	
K^+^ (mmol/L)	mean ± SD	4.37 ± 0.29	4.48 ± 0.55	0.702
	range	3.7–5	3.5–6	
**Urine pH**	mean ± SD	5.24 ± 0.6	5.47 ± 0.8	0.631
	range	5–7	5–7	
**Pathologic staging (TNM)**				
T1	n (%)	–	20 (41.7)	–
T2	n (%)	–	15 (31.3)	–
T3	n (%)	–	13 (27)	–
**Histological grading**				
G1	n (%)	–	13 (27)	–
G2	n (%)	–	23 (48)	–
G3	n (%)	–	12 (25)	–
**Angioinvasion**	n (%)	–	14 (29.2)	–

* ccRCC = clear cell renal cell carcinoma; WBC = white blood cells; LYM = lymphocytes; MONO = monocytes; NEU = neutrophils; EOS = eosinophils; BASO = basophils; INR = international normalized ratio; APTT = activated partial thromboplastin time; T-category of TNM classification T1 = tumour < 7 cm or less in greatest dimension, limited to the kidney; T2 = tumour > 7 cm in greatest dimension, limited to the kidney; T3 = tumour extends into major veins or perinephric tissues but not into the ipsilateral adrenal gland and not beyond Gerota fascia; G1 = low grade, G2 = moderately grade, G3 = high grade.

**Table 2:** Markers of oxidative stress in healthy controls and participants with ccRCC.

	Healthy Controls	ccRCC	p
AOPP (nmol/mg protein)	272.12 ± 134.5	300.28 ± 166.9	0.485
ACB (ABSU)	0.432 ± 0.07	0.44 ± 0.06	0.968
TAC (ABTS^•^, μmol TE/L)	284.65 ± 16.5	269.29 ± 11.7	0.0005
TAC (FRAP, μmol TE/L)	207.19 ± 45.9	210.56 ± 36.4	0.625
MDA (μmol/L)	3.28 ± 0.35	3.54 ± 0.54	0.096
4-HNE (pg/mL)	397.35 ± 159.36	445.11 ± 168.21	0.441

**Table 3:** Markers of oxidative stress in participants with ccRCC depending on the pathological stage.

	T1	T2	T3	*p*
AOPP (nmol/mg protein)	214.31 ± 55.1	253.88 ± 60.9	449.61 ± 216	0.013
Thiol groups (mmol/L)	645.51 ± 87.6	533.43 ± 80.3	441.99 ± 81.7	0.004
Amadori products (nmol/mg protein)	2204.91 ± 298.5	2557.43 ± 415.2	2576.35 ± 689.4	0.015
ACB (ABSU)	0.42 ± 0.01	0.43 ± 0.03	0.43 ± 0.06	0.638
3-nitrotyrosine (nmol/mg protein)	0.19 ± 0.012	0.2 ± 0.021	0.24 ± 0.037	0.017
Nitrate/nitrite (μM)	46.63 ± 11.2	63.76 ± 15.8	76.5 ± 21.5	0.07
TAC (ABTS^•^, μmol TE/L)	277.93 ± 6.8	265.02 ± 6.7	255.19 ± 5.2	<0.001
TAC (FRAP, μmol TE/L)	222.17 ± 35.4	219.19 ± 31	189.89 ± 33.9	0.204
MDA (μmol/L)	3.34 ± 0.5	3.71 ± 0.4	3.69 ± 0.7	0.178
4-HNE (pg/mL)	362.11 ± 138.8	404.88 ± 176.8	483.65 ± 142.1	0.081

**Table 4:** Markers of oxidative stress in participants with ccRCC depending on the RCC histological grade.

	G1	G2	G3	*p*
AOPP (nmol/mg protein)	224.75 ± 54.9	314.86 ± 179.8	423.05 ± 253.1	0.296
Thiol groups (mmol/L)	599 ± 110	522.53 ± 55.2	403.36 ± 54.4	0.003
Amadori products (nmol/mg protein)	2343.24 ± 291.9	2355.72 ± 492.9	2663.3 ± 555.4	0.525
ACB (ABSU)	0.42 ± 0.04	0.44 ± 0.05	0.44 ± 0.04	0.946
3-nitrotyrosine (nmol/mg protein)	0.21 ± 0.027	0.2 ± 0.024	0.24 ± 0.046	0.149
Nitrate/nitrite (μM)	51.9 ± 18.4	65.74 ± 18.7	74.66 ± 22.7	0.218
TAC (ABTS^•^, μmol TE/L)	276.92 ± 9.7	267.52 ± 11.7	257.99 ± 5.5	0.051
TAC (FRAP, μmol TE/L)	215.63 ± 37.7	215.49 ± 38.2	180.67 ± 11.5	0.189
MDA (μmol/L)	3.28 ± 0.3	3.29 ± 0.3	4.18 ± 0.5	0.008
4-HNE (pg/mL)	394.26 ± 101.1	383.15 ± 166.4	485.99 ± 192.7	0.657

**Table 5:** Markers of oxidative stress in participants with ccRCC with and without angioinvasion.

	ccRCC with Angioinvasion	ccRCC without Angioinvasion	*p*
AOPP (nmol/mg protein)	345.02 ± 190.6	282.38 ± 160	0.228
Thiol groups (mmol/L)	467.9 ± 95.9	536.04 ± 118.2	0.33
Amadori products (nmol/mg protein)	2504.86 ± 422.5	2351.75 ± 448.6	0.726
ACB (ABSU)	0.43 ± 0.06	0.43 ± 0.03	0.459
3-nitrotyrosine (nmol/mg protein)	0.22 ± 0.04	0.2 ± 0.022	0.483
Nitrate/nitrite (μM)	83.95 ± 17	58.09 ± 18.4	0.012
TAC (ABTS^•^, μmol TE/L)	255.54 ± 4.5	274.24 ± 8.7	<0.001
TAC (FRAP, μmol TE/L)	208.51 ± 39.8	211.38 ± 36.4	0.668
MDA (μmol/L)	4.28 ± 0.5	3.48 ± 0.3	0.002
4-HNE (pg/mL)	540.05 ± 185.2	404.42 ± 149.1	0.149

## Data Availability

All data included in this study are available upon request by contact with the corresponding author.
